# Micro Electrical Discharge Machining of Ultrafine Particle Type Tungsten Carbide Using Dielectrics Mixed with Various Powders

**DOI:** 10.3390/mi13070998

**Published:** 2022-06-25

**Authors:** Sai Dutta Gattu, Jiwang Yan

**Affiliations:** Faculty of Science and Technology, Keio University, Yokohama 223-8522, Japan; gattu.46@keio.jp

**Keywords:** micro-EDM, tungsten carbide, carbon nanofiber, silicon, alumina

## Abstract

Electrical discharge machining (EDM) is widely used to machine hard materials, such as tungsten carbide; however, the machining rate and surface quality are low. In this research, the effects of mixing electrically conductive carbon nanofiber (CnF), semiconductive silicon (Si) powder, and insulative alumina powder (Al_2_O_3_) at different concentrations in a dielectric fluid were studied by observing single discharge craters and hole machining performance in the EDM of ultrafine particle type tungsten carbide. Craters obtained using carbon nanofiber and alumina were much smaller than in oil-only conditions. In contrast, The results show that adding CnF significantly improved the material removal rate under all conditions. Si and Al_2_O_3_ powders only improved the machining performance at a high discharge energy of 110 V. Furthermore, improvement in surface roughness was observed prominently at high voltages for all the powders. Among the three powders, alumina was found to improve the surface roughness the most.

## 1. Introduction

Tungsten carbide (WC) and its composites (WC-Co) possess excellent physical properties, such as high melting and boiling points, wear, and corrosion resistance. As a result, it is useful in various industrial applications, notably in die/mold making, cutting, and surgical tools. Consequently, various studies have focused on machining tungsten carbide economically with high precision. Conventionally, tungsten carbide is machined using turning and grinding [1,2,3] using PCD and CBN tools. However, the tool wear is high, increasing costs [4]. In recent years, there has been a growing trend of using non-conventional methods, such as electric discharge machining (EDM) [5], electrochemical machining (ECM) [6], and laser machining [7], to process tungsten carbide products.

EDM is a thermo-electric process that removes material by thermal erosion due to repeated high-energy electrical sparks. Since there is no physical contact between the workpiece and electrode in this process, the machinability depends only on the tool and the workpiece’s electrical and thermal properties. Thus, this process can be used to machine any given conductive material regardless of its hardness. In addition, this method can also be used to directly transfer complex 3D shapes with high precision onto the workpiece without force-induced deformation.

Consequently, various studies have been performed on the EDM of tungsten carbide. Studies by Hourmand et al. [8] and Assarzadeh et al. [9] have used analytical models, such as response surface methodology and ANOVA analysis, to determine the optimum parameter values of peak current, spark time on and off, and gap voltage. Research on the effect of EDM on hardness and sub-surface damage on tungsten carbide has also been performed [10]. Furthermore, Jahan et al. [11] have studied the effect of different electrodes on EDM efficiency by comparing the material removal and electrode wear rate. However, it has been found that the EDM of WC-Co has low machining efficiency, with a high tool wear rate and surface defects. Recently, using tool rotation or vibration [12,13], servo movement and flushing strategies [14,15], and adding the powder to the EDM of WC-Co, have been studied to improve these shortcomings.

The dielectric separating the electrodes serves many functions during the EDM process. In addition to insulation, it helps transport debris away and acts as a coolant during the discharge phenomenon [16]. It has been studied that adding the powder to the dielectric during the EDM process can alter the machining process by dispersing the discharges and thus, improving machining efficiency [17]. Various studies have shown that powder addition improved material removal rate, electrode wear rate, and surface roughness. Furthermore, a few studies also focused on optimizing the powder-mixed EDM (PMEDM) of WC-Co using analytical models [18,19]. Jahan et al. [20] have also developed a mathematical model to study powder behavior during EDM. Their model underlines that the presence of any additive within the dielectric results in an increase in the discharge gap.

The most commonly used powders in EDM are aluminum (Al) and graphite (Gr). Their high electrical conductivity, low density, availability of particles on a nanometer scale, and low hazardous nature allow them to be widely used. However, in recent years, the effects of many other particles, such as silicon (Si), graphene, molybdenum sulfide (MoS_2_), and alumina (Al_2_O_3_) have also been studied. Pecas et al. [20] have shown that under a few conditions (powder concentration of 2 g/L for 1 mm^2^ electrode), silicon powder addition can generate a mirror surface finish on AISI H13 steel. Liew et al. [21] have used carbon nanofiber-assisted EDM of reaction-bonded silicon carbide and have significantly improved material removal and electrode wear rates. Sahu et al. [22] and Jahan et al. [23] have used nano-alumina powder in the EDM of Inconel and tungsten carbide, respectively. They have shown an increase in the material removal rate and a decrease in the surface roughness for specific parametric values.

Many studies have been performed on the EDM of tungsten carbide. However, up to date, the effects of using these newly attempted types of powders on the EDM of WC-Co, especially the ultrafine particle type WC-Co, which is a more promising material in the industry, are still unknown. In particular, the effect of powders in the micro-EDM process, where the discharge gap is much smaller than in conventional EDM is unclear. To evaluate the impact of characteristics of different powders, we studied the single discharge craters and their progression during EDM drilling. The finding of this study reveals the process mechanisms and parameters to improve the machinability of ultrafine particle-type tungsten carbide using micro-EDM.

## 2. Process Mechanisms of Powder-Mixed EDM

During EDM, an essential concern is the expulsion of debris outside the discharge gap. The debris generated during machining is generally removed by two mechanisms: bubble expansion and dielectric flow [12,16]. When the debris is not properly flushed out, it will result in (a) secondary discharges, where the debris is machined instead of the workpiece, or (b) short-circuiting. This results in frequent tool backtracking, increasing machining time, and increased costs. If the discharge gap widens, the dielectric can easily flow in, promoting the flushing of debris and improving process stability.

**Figure 1:**
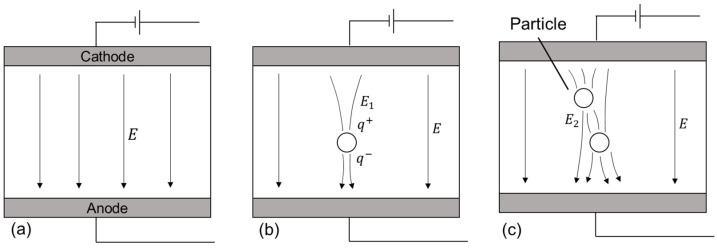
Schematic of (**a**) electric field in between two electrodes and (**b**) distortion due to presence of a single particle, (**c**) bridge formation with multiple particles.

When a particle is introduced in an electric field (*E*), as shown in Figure 1a, the polarization of the molecule occurs. This polarization results in an electric field aberration (*E_1_*) within the vicinity of the particle, as shown in Figure 1b. During EDM, when the electrodes are brought closer to each other, this aberration results in field intensification near the particle, creating a more accessible pathway for electrons to flow, thus, promoting dielectric breakdown. Moreover, when several particles get closer to each other, as shown in Figure 1c, the field is intensified further (*E_2_*), creating ‘bridges‘ for ions to flow, promoting even faster breakdown. As a result, the interelectrode gap required to form sparks is larger. Several studies [21,23,24] have reported that these promote process stability, thus improving machining efficiency.

Since the presence of particles alters the discharge characteristic, it is vital to study the effect of the properties of different powders. It has been reported that particles generally exhibit four different behaviors within the discharge gap: reciprocation, bridging, adherence to the electrode, and agglomeration [25]. Polarized molecules reciprocate between electrodes when an electric field is applied due to electromotive force. The physical and electrical properties of molecules determine the extent of this movement. At the same time, bridging occurs when particles align themselves parallel to the electric field, forming chain-like structures (Figure 1c). Reciprocation and bridging promote dielectric breakdown.

In contrast, the adhesion of particles on the workpiece surface inhibits the machining by preventing energy transfer. Moreover, it has been observed that nanoparticles tend to agglomerate due to high surface energy or Van der Walls forces. The particle agglomeration will decrease polarizability, inhibiting discharge channel formation and increasing the possibility of short-circuiting.

Carbon nanofibers are incredibly light and highly conductive materials. It has been reported that due to these properties, the nanofibers form bridges quickly, improving the machining output [21]. On the other hand, alumina is a dense material with very low electric conductivity. Due to these properties, the bridging of particles will not occur, and the sedimentation of the particles on the bottom surface will be prominent, inhibiting machining. Silicon is a semiconductor with a density and electrical conductivity between alumina and carbon nanofibers. Semiconductors exhibit a unique property of decreasing resistivity with an increase in temperature. Thus, depending on the condition within the working gap, silicon particles may exhibit all kinds of behaviors mentioned above.

Based on the process mechanism and powder behavior during EDM, the differences in the material removal, tool wear, and surface roughness during PMEDM of WC-Co are elucidated.

## 3. Experimental Methods

### 3.1. EDM Setup

**Figure 2:**
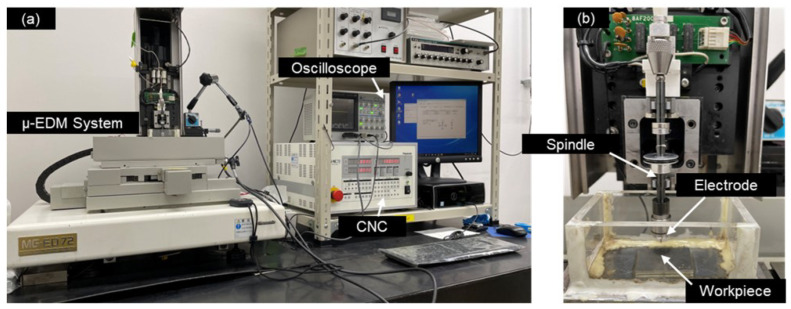
Photograph of (**a**) µ-EDM system, (**b**) electrode and workpiece arrangement.

In this study, a micro-EDM machine, Panasonic MG-ED72, was used. The device has a resolution of 0.1 µm in the x, y, and z axes. The machining is performed using a resistor-capacitor (RC) circuit, which is suitable for micromachining [26]. The control parameters are voltage ranging from ±50 V to ±110 V and capacitance of 3300, 1100, 200, and 100 pF. The discharge gap, pulse duration, and peak current are not controllable. A spindle is attached to the micro-EDM machine, rotating at 3000 rpm. An acrylic tank was fabricated to contain the dielectric. Discharge progress was monitored using an oscilloscope. The photograph of the machining setup is shown in Figure 2. During experimentation, no external stirring or circulation of the dielectric was applied.

### 3.2. Electrode and Workpiece

**Figure 3:**
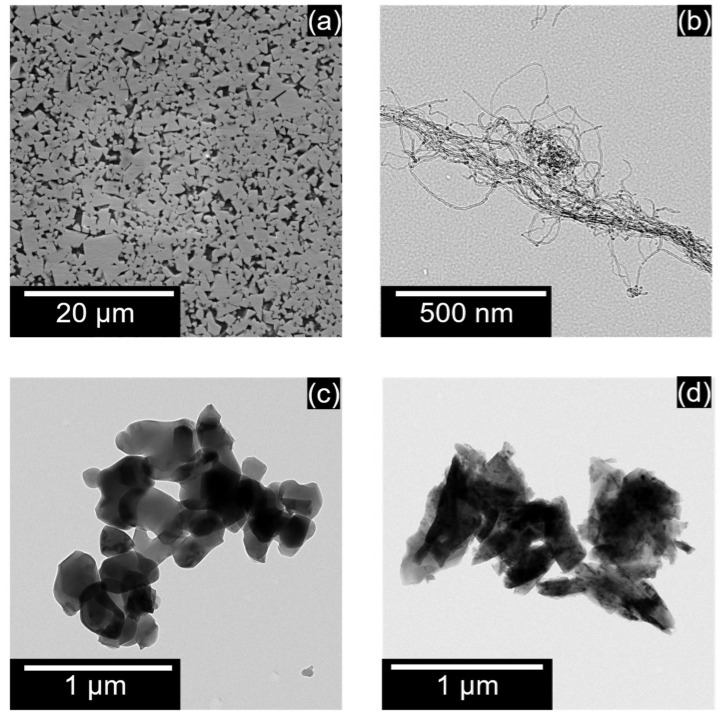
SEM of (**a**) tungsten carbide, TEM of (**b**) carbon nanofiber, (**c**) alumina, (**d**) silicon particles.

Ultra-fine grade cemented Tungsten carbide (WC-Co) KM-10, produced by Toyo Tool, Japan, was used as the workpiece material. The average grain size was around 0.7 µm, and the cobalt binder concentration was about 6%. The mechanical properties of the workpiece are shown in Table 1. The surface of the workpiece was mirror-polished to 0.01 µmSa surface roughness using a diamond powder slurry. A copper (Cu) electrode of a diameter of 1 mm from Nilaco Corporation, Japan, was used as a tool material. The electrodes were deburred and dressed using reverse-polarity EDM before each machining test. The scanning electron micrograph (SEM) of the surface of the workpiece is shown in Figure 3a.

### 3.3. Powder Materials

**Figure 4:**
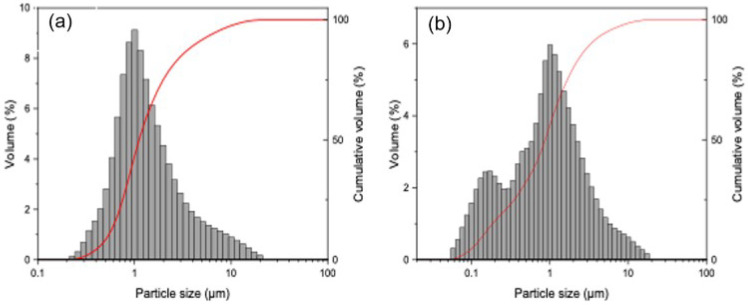
Particle size distribution of (**a**) silicon and (**b**) alumina used in this study.

Three different powders of varying shapes, densities and electrical properties were used in this experiment. Commercially available polishing grade alumina powder, Baikalox 1CR, manufactured by Baikowski International Co. Ltd., was used as the insulator powder. Semiconductive silicon powder, manufactured by Kyocera, Japan, was used. Lastly, carbon nanofibers of diameter 10–20 nm and height 0.1–10 µm, produced by Mitsubishi Chemicals, Japan, were used as a conductive powder in this experiment. The transmission electron micrographs [TEM] of carbon nanofiber, alumina, and silicon are shown in Figure 3b–d, respectively. A laser particle analyzer from Horiba semiconductors, LA-960, was used to measure the particle size. Figure 4a,b show the size distribution of the silicon and alumina powders, respectively. The thermal and electric properties of the powder are listed in Table 2.

### 3.4. Machining Conditions

Two different EDM tests were performed to determine the effect of powder addition on EDM: fundamental characteristics and hole machining tests. The micro-EDM machine used in this study uses an in-built contact detection program. An edge is detected if a threshold number of discharges occurs within a resistance-capacitance (RC) cycle. This method was used to obtain isolated single craters. Next, machining tests on the evaluation of PMEDM were performed by drilling holes of a depth [h] of 50 µm under different conditions, as shown in Table 3. The discharge energies used in this experiment are used for rough-phase micromachining. Full factorial experiments were performed with two repetitions for each condition. A hydrocarbon-based dielectric, Casty Lube EDS was used. As seen in Figure 3b–d, powders tend to agglomerate. Hence, the dielectric and powder mixture was subjected to an ultrasonic bath for 15 min before each machining to obtain a homogenous mixture.

### 3.5. Evaluation Conditions

The machining output of EDM with various powders was evaluated with three parameters: material removal rate, electrode wear rate, and surface roughness. Material removal rate (*MRR*) is defined as the ratio of the volume of the material removed (Vol_m_) to machining time (*M_t_*) Equation (1). A laser microscope from Olympus, Japan, was used to map the three-dimensional topography of the machined hole. The analysis software TalyMap, Amtek Corp. was used to determine the mean depth plane [*d*], and the diameter [*D*] at (z = 1/2 *d*) of the hole machined.

(1) $$MRR=\frac{\pi D^{2}d}{4\;M_{t}\;}$$

Electrode wear rate is defined as the ratio of the volume of electrode worn Vol_e_ to machining time (*M_t_*). Electrode wear is an essential factor in determining the economic feasibility of the machining process. Since the micro-EDM machine used in this study does not use any wear compensation mechanism, the difference between set depth [*h*] and actual machined depth [*d*] is the electrode wear. Since machining time varies based on machining conditions, studying the electrode’s relative wear to that of the workpiece is considered in this research. The equation to determine relative electrode wear rate (*REWR*) is shown in Equation (2). The machined region’s average surface roughness (Sa) was measured over an area of 256 × 256 μm at intervals of 0.25 μm. The ISO 25178 standard of measurement with no filters, measured over three locations on each machined surface was performed.

(2) $$REWR=\frac{D_{e}{}^{2}\left[h-d\right]}{D^{2}d}$$

## 4. Results and Discussion

### 4.1. Single Discharge Experiments

**Figure 5:**
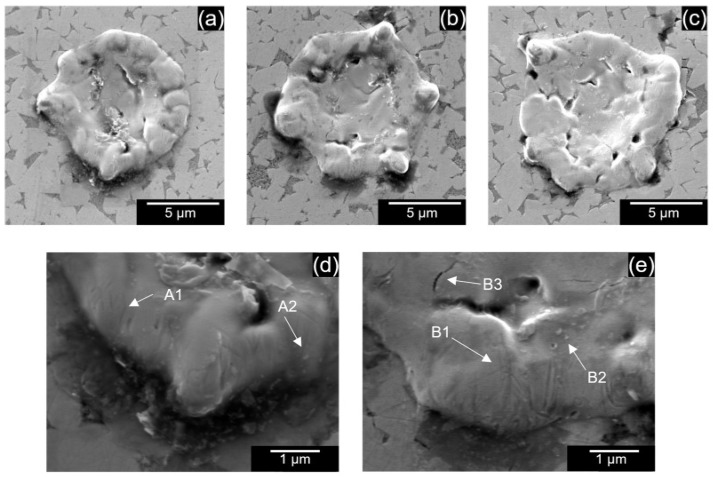
Single discharge craters for (**a**) 90 V, (**b**) 100 V, (**c**) 110 V using EDM oil without powder addition. Crater edge at (**d**) 90 V, (**e**) 100 V.

Figure 5a–c shows the single discharge craters obtained using EDM oil without powder addition for 90, 100, and 110 V, respectively. Unlike the craters in the EDM of other materials, such as silicon carbide [12,21,27], which show clear circular boundaries, craters in WC-Co were irregular. Furthermore, enlarged images of the crater surface, as shown in Figure 5d,e, indicate that the machining has not completely progressed to the crater’s edge since the polishing marks from the original sample are retained after EDM (A1 and B1). These marks indicated that at these discharge energies, the heat generated is not sufficient to melt the material at the edges; instead, the grains are pushed outwards. Additionally, as seen in A2–B2, redeposited materials are observed. Since tungsten carbide is a highly dense material, it is difficult to eject the molten materials entirely from the discharge gap, which redeposits onto the surface. The formation of micro-cracks was also observed, as seen in B3. Crack formation is due to the preferential removal of cobalt binder, which has a lower melting point than tungsten carbide, forming voids, which develop into cracks.

**Figure 6:**
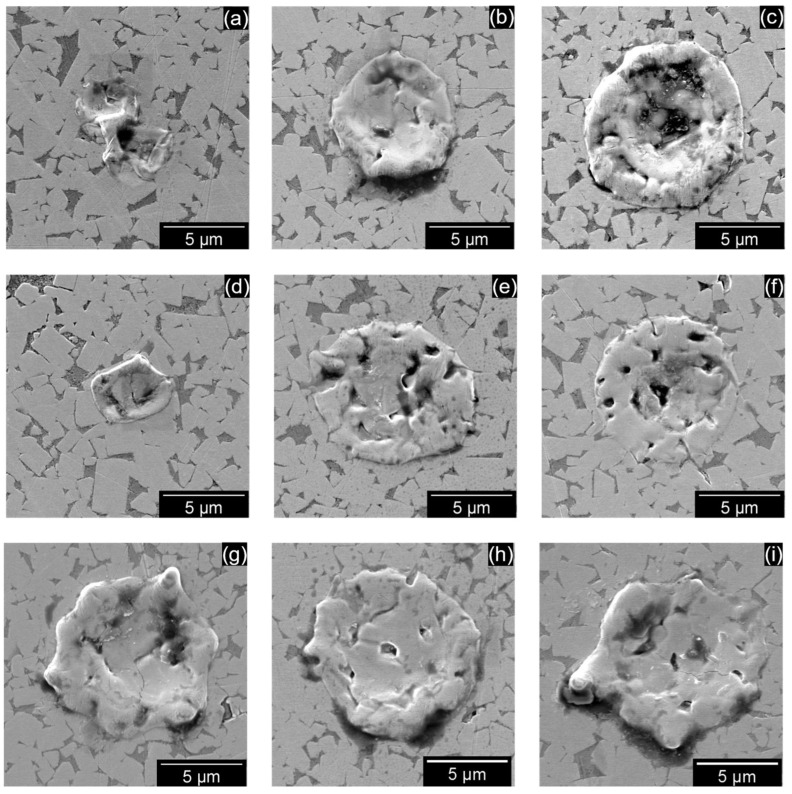
Single discharge craters for (**a**) 90 V, (**b**) 100 V, (**c**) 110 V using EDM oil with carbon nanofiber powder, (**d**) 90 V, (**e**) 100 V, (**f**) 110 V with alumina powder and (**g**) 90 V, (**h**) 100 V, (**i**) 110 V with silicon powder.

**Figure 7:**
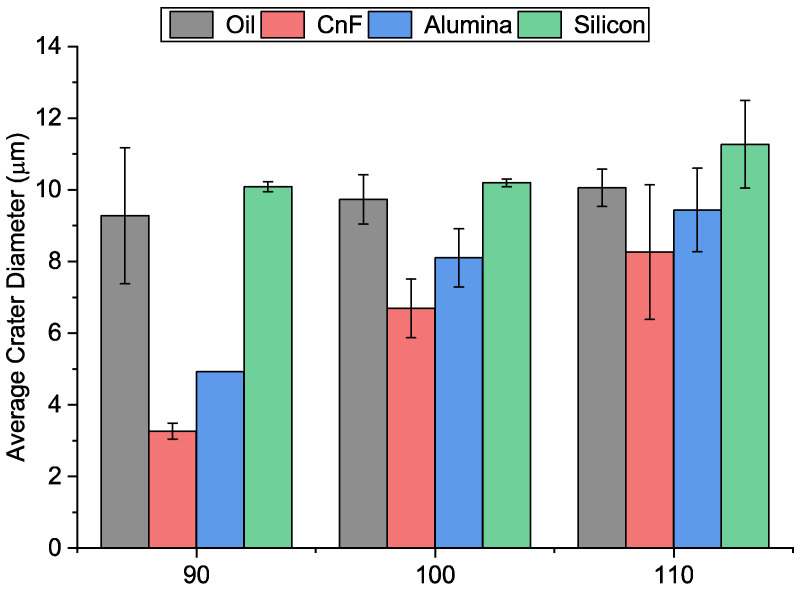
Average single discharge crater diameter under different conditions.

Figure 6a–i show the SEM images of single discharge craters observed at various PMEDM conditions. The average diameter of these craters is summarized in Figure 7. In the case of carbon nanofiber and alumina mixed EDM, the average crater diameter reduced, whereas it increased in the case of silicon. In addition, the crater diameter increased with voltage for all conditions.

According to previous research, the discharge gap increased drastically when the carbon nanofibers were added to the dielectric [21]. Furthermore, as explained earlier, the breakdown strength is significantly reduced due to its high conductivity. The plasma, which is formed, has lower energy; as it travels for a longer distance, it weakens further. This results in a narrow crater. In the case of alumina-mixed EDM, the increase in the discharge gap is not significant due to its low electrical conductivity. Additionally, alumina particles absorb plasma energy due to their high thermal conductivity. Since lower energy is transmitted to the workpiece, lower material is removed. However, in the case of silicon, contrary results were observed. Silicon exhibits thermal and electrical properties in between carbon nanofiber and alumina. Although the discharge gap is increased compared to the no powder condition, it is not as large as carbon nanofibers. As a result, an expanded plasma channel is formed, which results in a wider crater. Thus, it can be seen that the properties of powder influence the discharge process significantly.

### 4.2. Hole Machining by Carbon Nanofiber-Mixed EDM

**Figure 8:**
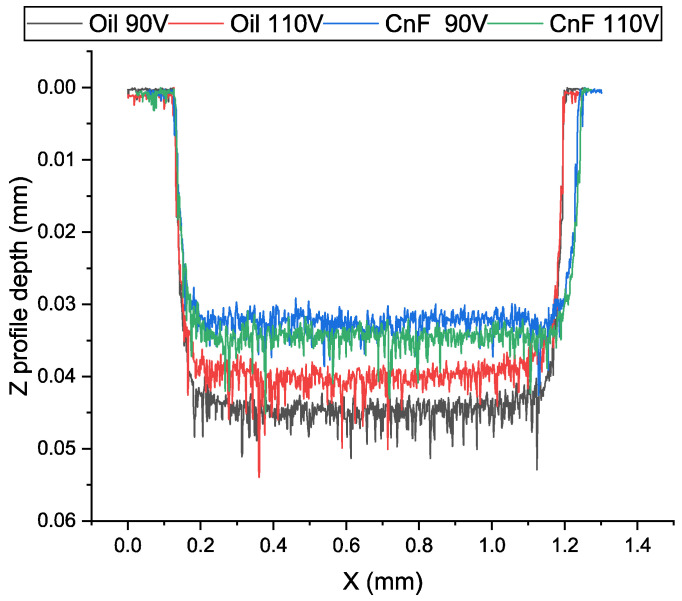
Profiles of machined holes at 90 and 110 V using oil and 0.5 g/L CnF-mixed EDM.

**Figure 9:**
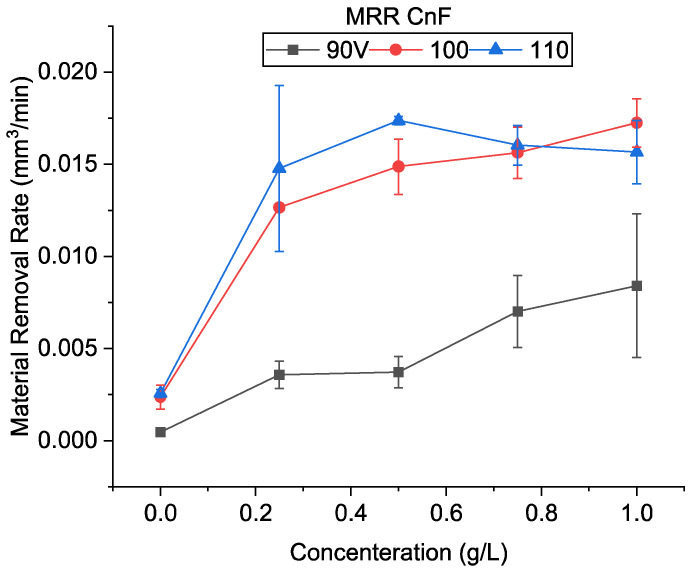
Material removal rate using CnF-mixed EDM.

The machined profiles and the material removal rate with oil and carbon nanofiber-mixed EDM are summarized in Figure 8 and Figure 9, respectively. In the only EDM oil condition, the maximum MRR obtained was 0.0026 mm^3^/min at 110 V condition, which decreased with the decrease in voltage. With an increase in voltage (*V*), the maximum discharge energy per spark (W) increases, and as a result, the material removed per discharge also increases, Equation (3). This can be seen evidently in Figure 7, where the crater diameter increased with an increase in voltage.

(3) $$W=\frac{\;1}{2}CV^{2}$$

It can be seen from the machined profiles in Figure 8 that the machined holes were shallower and wider with the addition of the carbon nanofiber. The increase in machined hole diameter, called overcut, is due to an increase in the discharge gap due to the addition of nanofibers.

**Figure 10:**
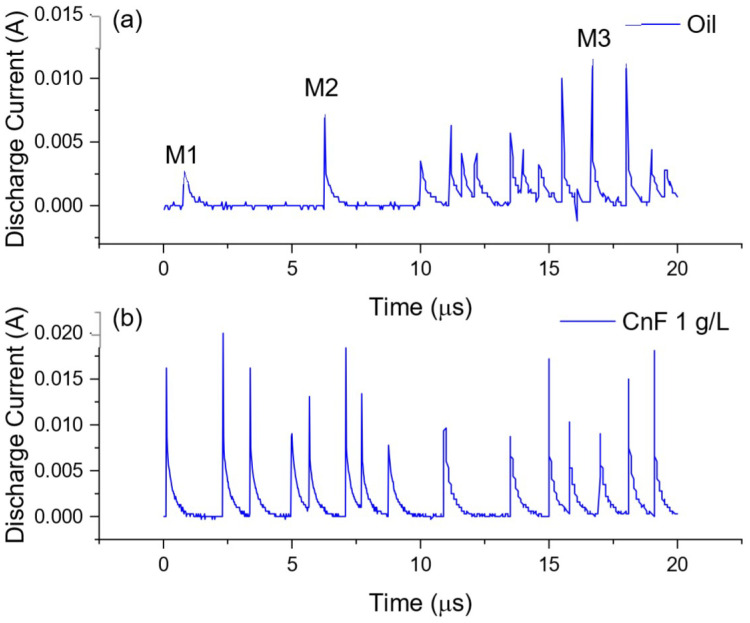
Current waveforms during EDM using (**a**) oil and (**b**) carbon nanofiber mixed EDM (1 g/L) at 110 V.

It was observed that even though the machined depth with the oil-only condition was large, the MRR increased for all concentrations of CnF and voltages. At a higher discharge energy of 110 V, the maximum MRR observed was 0.017 mm^3^/min at a concentration of 0.5 g/L, over 6.5 times that of the oil-only condition. This tremendous improvement in MMR can be attributed to the ease of discharge initiation and increased discharge gap width. It was also observed that at 110 V, the machining speed was equal to the set feed speed (0.5 µm/s) at all concentrations. This indicates no ineffective discharge period in this condition, where the tool is forced to retract to prevent short-circuiting, could be observed. This can be monitored by observing the waveforms on the oscilloscope, as seen in Figure 10a. It was observed that during EDM with oil, there was a lot of arcing which had the waveform M1 in contrast to normal EDM (M2). It was also observed that if arcing persisted, it led to the formation of complex waveforms [28] as seen in M3, followed by short-circuiting and tool retraction. In contrast, as seen in Figure 10b, the addition of carbon nanofiber increased the number of sparks, and complex waveforms were not observed.

It can be seen from Figure 10 that the MRR increases with an increase in concentration and then decreases after a peak at 0.5 g/L. In EDM with oil without process alteration, it has been reported that the spark tends to accumulate in one region [12,29]. As debris concentration increases, spark formation in the vicinity is likely, as shown in Figure 1b. Whereas, in powder-EDM, sparks are not localized since particles are dispersed throughout the surface. In CnF-mixed EDM, initially, as the particle concentration increases, there is an increase in the number of discharge pathways dispersing EDM, increasing process stability, and thus increasing MRR. However, as the particles’ concentration increased, many nanofibers started to either accumulate or adhere to the electrodes, inhibiting the discharge process.

Similarly, an improved machining efficiency was observed when the voltage was reduced to 90 V. A machining rate of 0.008 mm^3^/min was achieved, over 18 times that of only oil. Generally, the discharge gap decreases considerably at low voltages, increasing the possibility of debris accumulation within the working gap. This increases the frequency of ineffective discharges, such as short-circuiting due to debris accumulation and secondary discharges on the machined debris. Thus, flushing away debris becomes a prime concern. During CnF-mixed EDM, the machined speed increased from 0.18 to 0.53 times the set machining speed when the concentration increased from 0.25 to 1.0 g/L. There was a significant no discharge period. However, during the tool backtracking period, the fresh dielectric with powder flows in, removing the debris. This period is essential at low voltage, where the discharge gap is lower, as it resets the dielectric. The proportion of powder entering the gap is higher at higher concentrations so that machining can be restarted quickly. Although the machining efficiency increased, it can be considered that at a feed rate of 0.5 µm/s and 90 V using carbon nanofibers, uninterrupted machining cannot be achieved entirely.

An improvement of over 7.7 times in the MRR without powder addition was observed at 100 V. Similar to 110 V, the machining was almost continuous, the machining speed increasing from 0.68 to 0.92 times the feed speed. Furthermore, the MRR increased with concentration. At 1.0 g/L, the MRR was even higher than 110 V. The brief intermissions during EDM at 100 V allowed new dielectrics to flow in, which is considered to improve the machining rate compared with 110 V.

**Figure 11:**
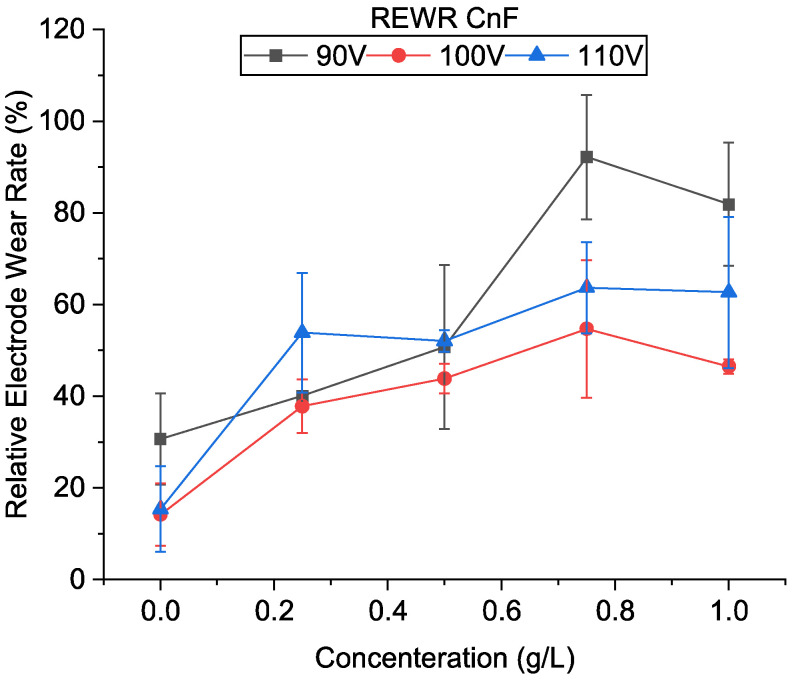
Relative electrode wear rate using CnF-mixed EDM.

Figure 11 compares electrode wear for different machining conditions in CnF-mixed EDM. It was observed that, unlike in previous studies, electrode wear rate increased with the addition of CnF to the dielectric, which increases further with the increase in the powder concentration.

During the EDM process, plasma travels from the cathode and bombards the anode, removing material by heat transfer and abrasive action. However, some electrons also travel in the opposite direction removing material from the anode. Since the kinetic energy of ions is higher than electrons, the amount of material removed from the anode is much higher. In addition, the temperature in the discharge gap can exceed 3000 K [30], removing material from both electrodes. Thus, the tool wear depends on the electrodes’ electrical and thermal properties. The melting point of copper is much lower than that of tungsten carbide [1631 K], and thus, the melting of copper will be higher at high temperatures. At a high voltage or discharge energy, the fraction of electrons is high, and the temperature in the working gap is enormous. Hence a higher wear rate is observed. The dielectric flow into the discharge gap, which acts as a coolant and reduces the heat transferred to the electrodes is necessary to prevent electrode wear.

As mentioned above, the machining at higher voltages [100 and 110 V] was almost continuous. Since no external flushing was used, if no electrode backtracking occurs, the flow of the new dielectric is limited. The debris removed is only due to bubble explosion and not debris flow, and as a result, the temperatures within the gap will continue to rise. The electrode wear will increase correspondingly with an increasing machining rate, as seen in Figure 11. Since the discharge energy at 100 V is smaller than 110 V, the temperature reached within the gap is expected to be smaller, resulting in a lower tool wear rate.

In contrast, very high electrode wear was observed at 90 V. This result is similar to previous studies, in which the electrode wear rate decreased with the discharge energy and then increased. Due to improper flushing at lower energies, arcing will occur, which results in more material being removed from the electrode than the workpiece. A considerable variation was also observed in the relative electrode wear rate due to this instability, which is more prominent at 90 V. In addition to arcing, with an increase in concentration, the tool wear rate also increased due to continued machining similar to higher energies. A decrease in wear rate was observed at 1 g/L at all voltages. It is believed that at a very high concentration, adherence of particles to the surface of the electrode is increased. This adherence may have prevented the heat from being transferred from the dielectric to the electrode.

**Figure 12:**
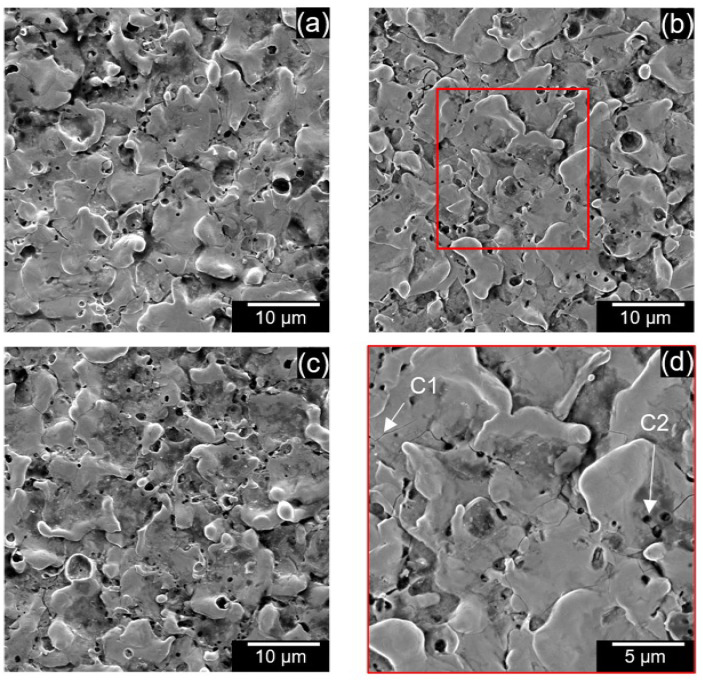
SEM of the machined surface using oil-only at (**a**) 90 V, (**b**) 100 V, (**c**) 110 V, and (**d**) enlarged region of 100 V.

**Figure 13:**
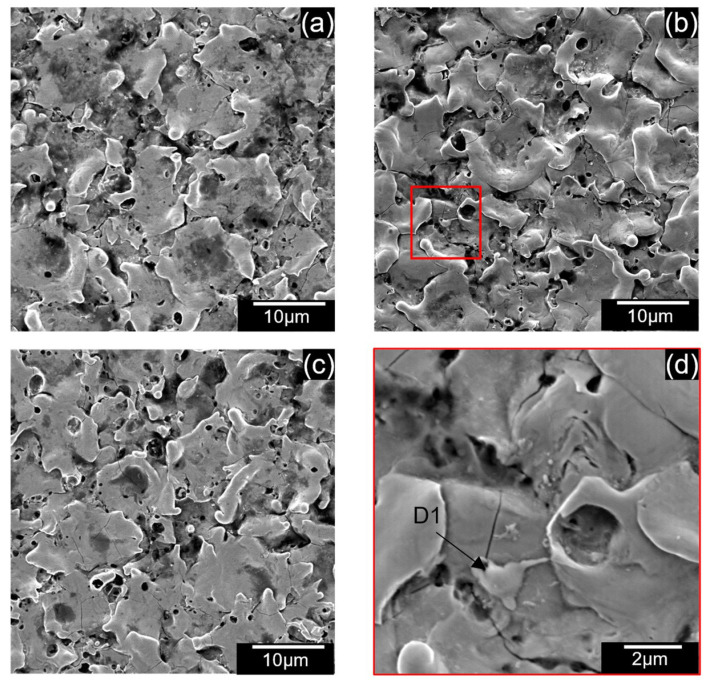
SEM of machined surface using 0.5 g/L carbon nanofibers at (**a**) 90 V (**b**) 100 V, (**c**) 110 V, (**d**) enlarged region of 100 V.

Figure 12 shows the SEM of the surface of the machined holes at different voltages using EDM oil. The craters are narrow and shallow at lower voltages (Figure 12a,b). Many surface defects, such as micro-cracks (C1), micro-pores (C2), and redeposited materials (C3) were observed on the surface. The number of redeposited materials increased at higher voltages, as seen in Figure 12c. At high voltages, the gap temperatures were larger, and molten debris could not be cool down quickly, so they redeposit onto the surface. Similar surface defects could also be seen in carbon nanofiber-mixed EDM as seen in Figure 13a–d. However, the redeposited materials were more extensive (D1) and could also be seen at lower voltages Figure 13a,b.

**Figure 14:**
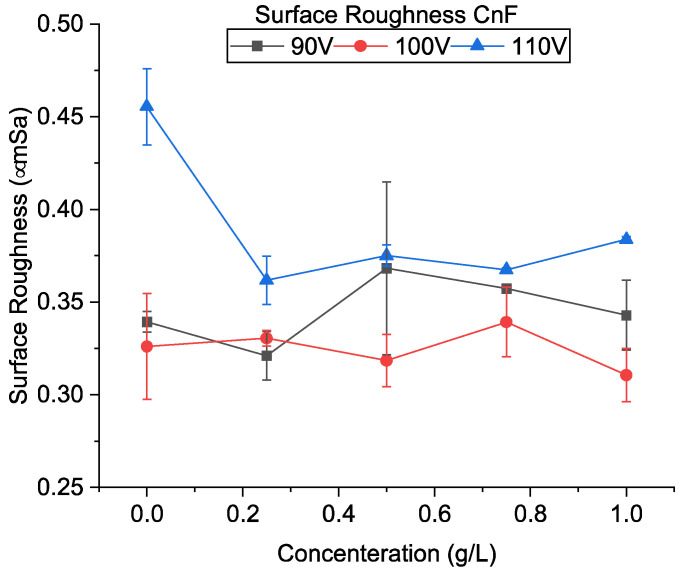
Surface roughness using CnF-mixed EDM.

Previous results have indicated that adding powder increases discharge dispersion, creating a uniform surface. In addition, it has also been reported that using powder-mixed EDM produces wide yet shallow craters [31]. These two factors are considered to result in lower surface roughness. Figure 14 shows the result of surface roughness. At 110 V, adding powder decreased the surface roughness for all concentrations, whereas the surface roughness has shown various trends for lower voltages.

At higher discharge energy, the material removed per discharge is increased. This results in a deep crater, as seen in Section 4.1. As the discharge energy decreases, the crater size decreases, resulting in a smoother surface finish. This phenomenon is also observed in this study when the voltage is reduced from 110 to 100 V. However, the surface roughness increases when the voltage is further reduced. At the 90 V condition, redeposited materials could have increased surface roughness when compared to only the EDM oil condition, as seen in Figure 13a.

In summary, using carbon nanofiber powder-mixed EDM of tungsten carbide improved the machining rate significantly under all machining conditions. However, an increased tool wear rate was observed. Under certain machining conditions, a fine surface was observed. Among the machining conditions, at 100 V, a high material removal, low tool wear, and a smoother finish can be simultaneously achieved at concentrations of 0.5–0.75 g/L. These results also agree with a previous study [21], which showed improvement in the MRR using carbon nanofiber on reaction-bonded silicon carbide.

### 4.3. Hole Machining by Alumina-Mixed EDM

**Figure 15:**
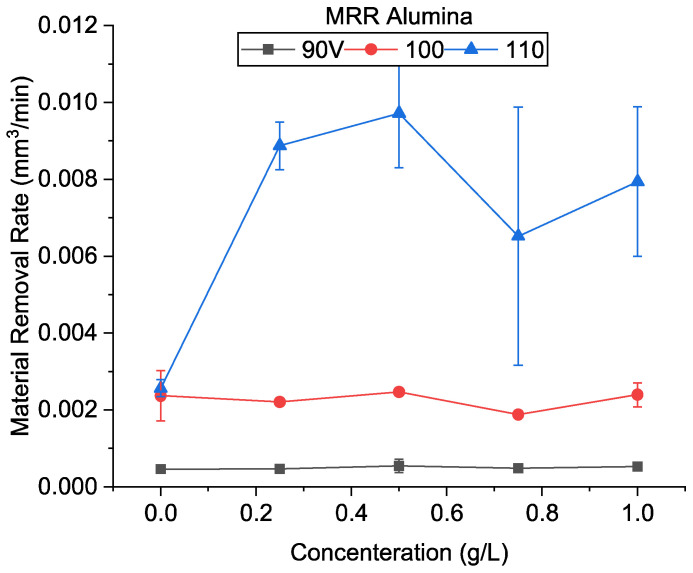
Material removal rate using alumina-mixed EDM.

Figure 15 shows the MRR obtained at various machining conditions using alumina. At high discharge energy, similar to CnF, there was a higher MRR when compared to the no powder condition. The highest MRR obtained was 0.00971 mm^3^/min at 0.5 g/L, about 3.8 times of the oil-only condition. It was also observed that the MRR increases initially and then decreases with an increase in concentration. As mentioned in Section 4.2, it has been proven theoretically and experimentally that particle addition increases the discharge gap regardless of the particle’s properties. However, unlike CnF, due to its insulating nature, the alumina particles do not get polarized easily in an electric field due to the absence of free electrons, and dipoles are not created. The formation of ‘bridges‘ is inhibited. However, under strong electric fields, even insulative materials become polarized. This indicates that the alumina particles can participate in spark formation at high voltages. This effect reduces as the voltage is reduced. These results agree with previous studies in which alumina has been shown to improve the MRR in transistor circuits at high peak voltages [22,32]. However, in fine-finishing micro-EDM using RC circuits, such as this study, the improvement in the MRR is not significant. Additionally, it was found that the machined feed speed varied between 2.32 at 0.5 g/L and 3.28 at 0.75 g/L to that of the set feed speed. These results show that although there is an improvement in the MRR at 110 V compared to oil, there was still a significant amount of time in which machining did not occur.

At the low discharge energy of 90 V, adding alumina increased the MRR at all concentrations. However, the improvement was not substantial. The highest MRR was 0.00055 mm^3^/min, 1.19 times the only oil condition. It can be seen from Figure 7 that the average crater size of alumina-mixed EDM was much lower than that of oil-only conditions at all conditions, indicating that the material being removed per spark is very low. Thus, the machining rate improvement is due to the lower no-discharge period. The machined feed speed was also similar to the no powder added condition. It was observed that, due to the higher density of alumina than the dielectric, the powder settling was a significant phenomenon. Since no external stirring or circulation was used, the powder sedimentation cannot be overcome.

Furthermore, as the machining time increased, the fraction of powder suspended in the dielectric decreased. During electrode backtracking, the fresh dielectric contained a limited amount of alumina. As a result, when machining time is extended, the MRR becomes similar to that of oil only. In addition, as mentioned earlier, the alumina addition does not directly improve spark formation. An increase in particle concentration will only inhibit the EDM process. Additionally, similar to that at 90 V, no significant improvement in the MRR was observed at 100 V.

**Figure 16:**
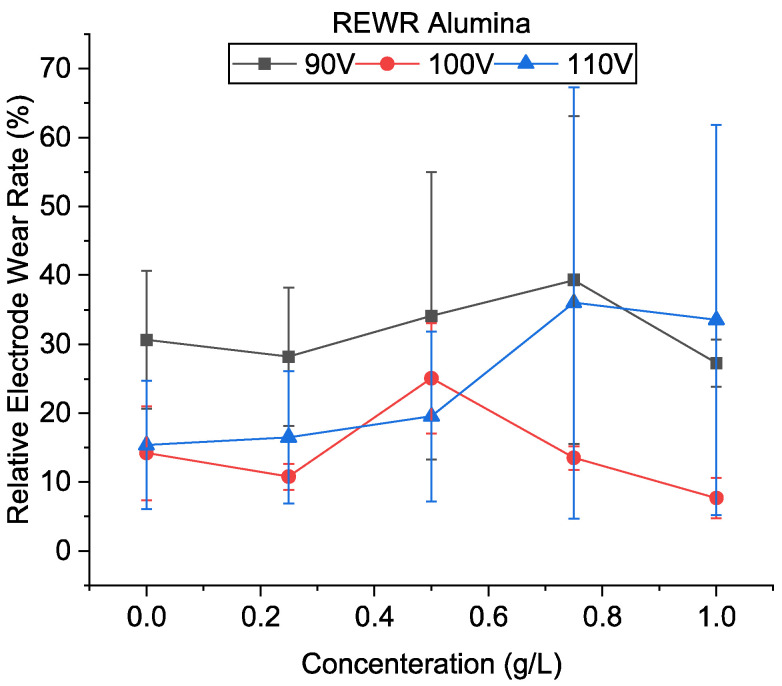
Relative electrode wear using alumina-mixed EDM.

Figure 16 shows the relative electrode wear of alumina mixed-EDM. Similar to CnF, the tool wear rate was generally found to increase with the addition of powder and was more significant at 90 V. Unlike CnF addition, where tool wear is due to high gap temperature due to continuous machining, the tool wear in alumina is expected to be due to frequent arcing and short-circuiting. As a result, a significant variation in the electrode wear results was observed. This is evident as electrode wear increases with a decrease in voltage and concentration for most conditions.

**Figure 17:**
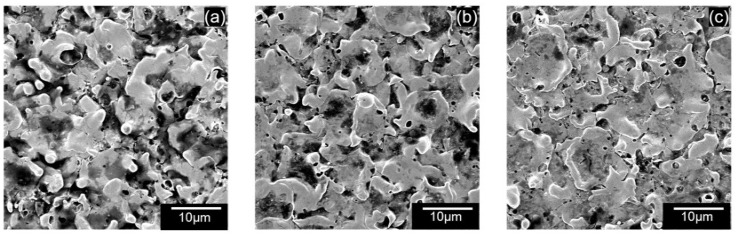
SEM of machined surface using 0.5 g/L alumina at (**a**) 90 V (**b**) 100 V, (**c**) 110 V.

**Figure 18:**
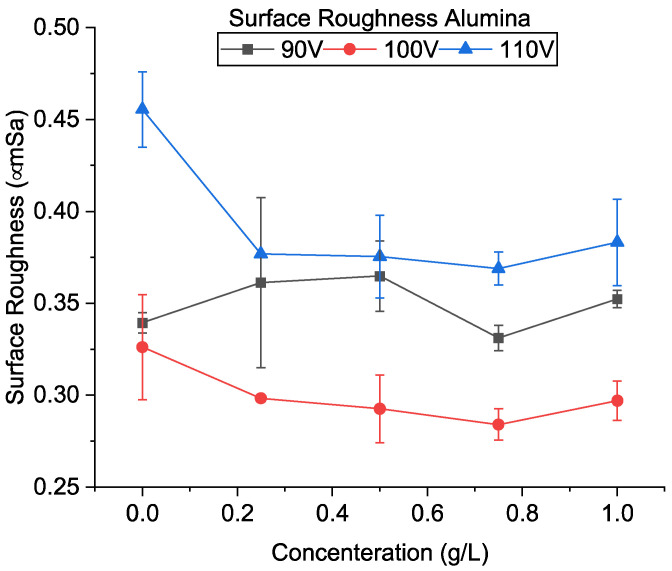
Surface roughness using alumina-mixed EDM.

Figure 17a–c shows the SEM of the machined surfaces with alumina mixing. The surface roughness of the machined holes using alumina is shown in Figure 18. The addition of alumina decreased the surface roughness for all concentrations at 110 and 100 V. Small and shallow craters, as seen in Figure 6d–f and uniform discharge distribution, helped to achieve a smoother surface. Moreover, as the voltage decreases to 90 V, frequent arcing and short-circuiting make the surface rough. It has also been observed that the effect of concentration does not significantly alter the surface roughness. As mentioned above, as the machining time increases, the powder concentration suspended within the dielectric becomes lower due to powder sedimentation, resulting in similar surfaces.

Effective machining using alumina can be achieved by using a high voltage of 110 V for rough machining and 100 V for fine finishing. The effect of concentration is not significant. In their study on using nano-alumina powders at finish phase machining [23], the authors indicated that there was no significant increase in the MRR but an improvement in surface roughness. In our study using micro-alumina at rough machining conditions, we found a similar roughness improvement. However, we have found that alumina can improve the material removal process at higher machining energies.

### 4.4. Hole Machining by Silicon Nanofiber-Mixed EDM

**Figure 19:**
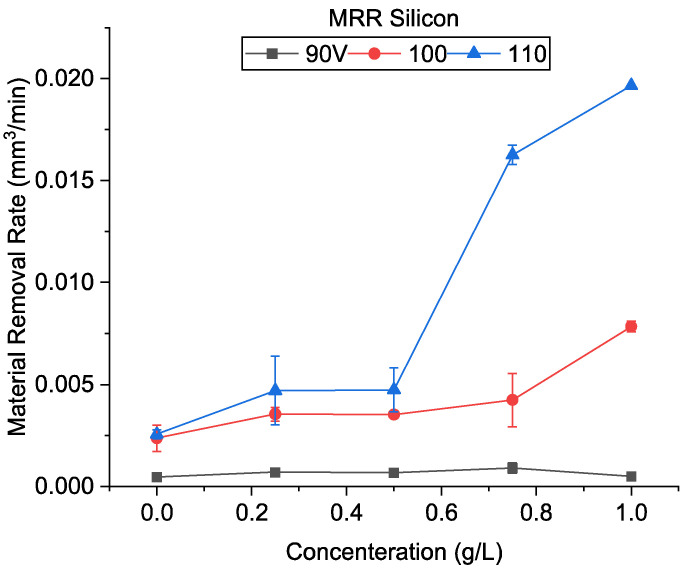
Material removal rate using silicon-mixed EDM.

The material removal rate obtained during the machining using silicon is shown in Figure 19. It was observed that the MRR increased for all conditions in the presence of silicon. The highest MRR of 0.0197 mm^3^/min was obtained at 1 g/L at 110 V. It was also observed that the MRR increases with an increase in particle concentration at higher voltages. Like CnF, silicon particles within the discharge gap cause electric field aberrations. However, silicon’s aberration is expected to be lower compared to nanofibers with very high electrical conductivity. Furthermore, silicon particles are much smaller than nanofibers that are 0.1–10 µm in length. Due to Van der Walls forces, particle agglomeration is also higher at smaller sizes.

As seen in Figure 7, the crater diameter of silicon, unlike CnF and alumina, is higher than that of oil. Additionally, the crater diameter did not vary significantly with voltage, similar to oil-only conditions. The material removed per discharge is thus higher than that of oil-only conditions. Moreover, the presence of silicon also improves the distribution of discharges. These two factors are expected to increase MRR.

Furthermore, semiconductors show a decrease in resistivity with an increase in temperature. Thus, if the machining is continuous and the working gap’s temperature increases, silicon’s conductivity would increase drastically. A highly conductive powder would further enhance the discharge breakdown for the following discharges, and the machining would proceed rapidly. The temperature in the working gap is generally higher at high discharge energies. Furthermore, an increase in concentration can enhance this effect. This is evident in the MRR results shown in Figure 19. It was observed that an increase in voltage increased the material removal rate. Furthermore, the increase in concentration improved it further.

However, at 90 V, the increase in concentration did not improve the MRR considerably and was found to be reduced at very high concentrations. At low voltage, the gap size is significantly smaller. The size of the agglomerated Si particles becomes similar to that of the gap itself. This inhibits the machining process, which reduces furthermore with an increase in powder concentration.

**Figure 20:**
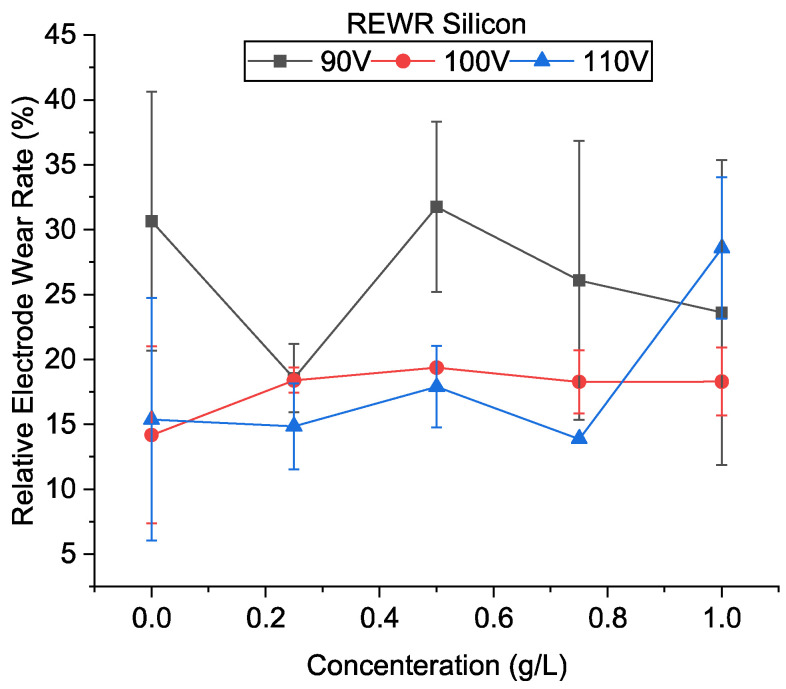
Relative electrode wear rate using silicon-mixed EDM.

The relative electrode wear for different machining conditions is shown in Figure 20. Like CnF and alumina, high tool wear was observed at 90 V, which decreased with an increase in voltage. It was due to the increased amount of arcing at low voltages. This instability in machining also resulted in a high fluctuation in electrode wear value at lower voltages.

**Figure 21:**
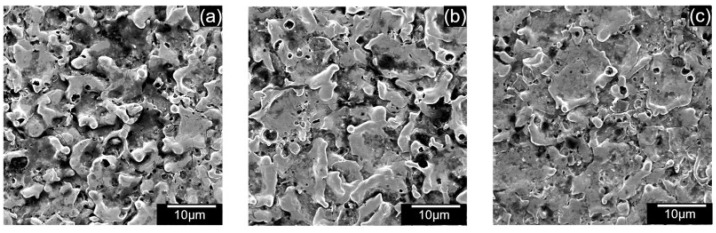
SEM of machined surface using 0.5 g/L silicon at (**a**) 90 V (**b**) 100 V, (**c**) 110 V.

**Figure 22:**
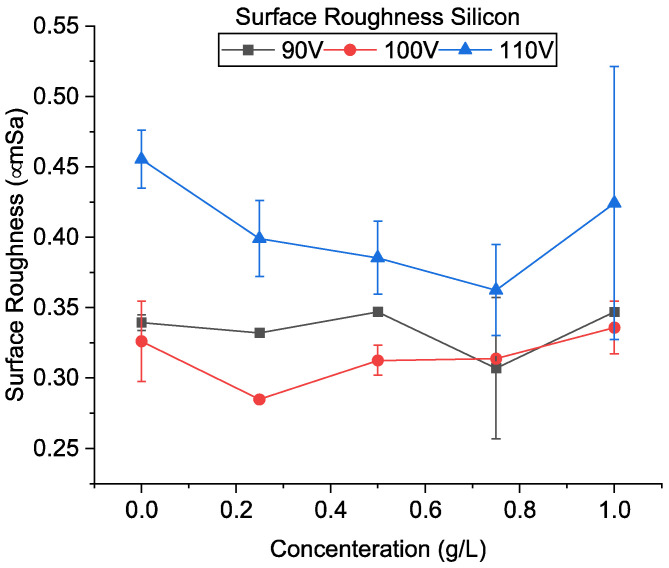
Surface roughness using silicon-mixed EDM.

Figure 21a–c shows the SEM of the machined surfaces with silicon mixing. Surface roughness is shown in Figure 22. It was observed that the surface roughness decreased for most conditions during PMEDM with Si powder. The roughness value decreases initially with voltage and then increases. Like CnF and Al_2_O_3_, the initial decrease in surface roughness was due to the reduction in crater size, as seen in Figure 7. However, unlike other powders, the difference in crater size between 90 V and 100 V is insignificant. The roughness increases due to higher proportions of short-circuits and arcing, causing unevenness throughout the surface.

Furthermore, it was observed that the roughness value decreases with increased concentration. Initially, with increases in concentration, the discharges become uniformly distributed due to a larger number of possible discharge pathways. However, at very high concentrations, many particles start to agglomerate, resulting in discharge concentration in the vicinity of the agglomerated particles and an uneven surface.

Silicon-mixed EDM of tungsten carbide can be an effective alternative to conductive powders. However, they can yield high material removal rates only at high discharge energy and powder concentrations. The electrode wear rate is also lower than CnF for most conditions. In their study, Pecas et al. [20] used silicon powder (10 µm) in the EDM of AISI H13. They have indicated that the addition of silicon improved the roughness values. We have also found that silicon addition can also improve the roughness in micro-machining conditions.

## 5. Conclusions

Three kinds of powders, conductive carbon nanofibers (CnF), semiconductive silicon (Si), and insulative alumina (Al_2_O_3_), were mixed in a dielectric fluid during electric discharge machining (EDM) of ultrafine particle type tungsten carbide. Single discharge and hole machining tests were performed, and their effects on material removal rate (MRR), relative electrode wear rate (REWR), and surface roughness were studied. When compared to conventional EDM without powder mixing, the following conclusions were made:

1. CnF and Al_2_O_3_-mixed EDM results in a crater with a smaller size when compared to oil EDM, which increases with an increase in voltage. Si-mixed EDM, like oil EDM, has large craters, which do not vary significantly with changes in voltage.
2. The MRR increased with the addition of all powders. Because of its electrical properties, CnF improved the MRR tremendously. Such effects were observed in Al_2_O_3_ and Si only at high voltages. The MRR increased initially, with an increase in concentration, then decreased.
3. A higher relative electrode wear rate (REWR) was also high using powder-mixed EDM. In particular, using CnF, large amounts of electrode wear were observed. Due to a smaller discharge gap at 90 V, an increased amount of process instability was observed and, as a result, higher electrode wear.
4. The surface finish was improved for all powders and concentrations at higher voltages of 100 and 110 V compared to EDM with oil only. However, such improvement was not observed when the voltage was reduced further, owing to much faster machining.
5. It was also found that the increase in powder concentration improved the surface roughness. Due to a much faster machining rate, carbon nanofibers produced a rougher surface compared to alumina and silicon.

At rough machining conditions, the objective is to achieve high machining material removal with repeatability. The results of this study indicate that conductive carbon nanofiber is the best choice for powder-mixed EDM of tungsten carbide since it can machine at a very high rate for all voltages and concentrations. Careful selection of machining parameters is required to obtain desired output using silicon and alumina powders. It is presumable that the addition of suitable powder could aid in improving the micro-processing of materials having similar microstructures to ultrafine particle tungsten carbide, whose machinability is lower.

## Figures and Tables

**Table 1:** Properties of tungsten carbide.

Property	Value
Material type	Ultrafine particle type (avg. size 0.69 µm)
Binder type and content ratio	Co (6%)
Density [g/cm^3^]	14
Melting point [K]	3140
Hardness (HRA)	91.5
Thermal conductivity [W/mK]	110

**Table 2:** Properties of different powders.

Property	Carbon	Silicon	Alumina
Density [g/cm^3^]	1.3–2	2.329	3.965
Electrical Conductivity [μΩ/cm]	50	1 × 10^5^	1 × 10^−9^
Thermal Conductivity [W/mK]	400	150	30
Melting Point [K]	3823	1687	2345

**Table 3:** Machining conditions.

Property	Value
Tool	Copper (Ø 1 mm)
Workpiece	WC-Co
Polarity	Tool (-ve)
Voltage [V]	90, 100, 110
Capacitance [pF]	3300
Feed rate [µm/s]	0.5
Powders	CnF, Al_2_O_3_. Si
Concentration [g/L]	0, 0.25, 0.5, 0.75, 1.0

## Data Availability

Not applicable.
